# Multiple Retinal Holes Secondary to Valsalva Retinopathy

**DOI:** 10.1155/2018/8950682

**Published:** 2018-05-08

**Authors:** Rajya L. Gurung

**Affiliations:** Vitreo-Retina Department, Biratnagar Eye Hospital, Biratnagar, Nepal

## Abstract

Valsalva retinopathy is a common condition but retinal holes secondary to Valsalva retinopathy are rarely reported. The author believes this to be the first report to describe multiple retinal holes after hyaloidotomy for Valsalva retinopathy.

## 1. Introduction

Valsalva retinopathy is a common condition but retinal holes secondary to Valsalva retinopathy are rarely reported. We report a case of Valsalva retinopathy with multiple retinal holes after YAG hyaloidotomy. To the best of our knowledge, this is the first reported case of multiple retinal holes in the posterior pole following YAG hyaloidotomy for Valsalva retinopathy.

## 2. Case Presentation

**Figure 1:**
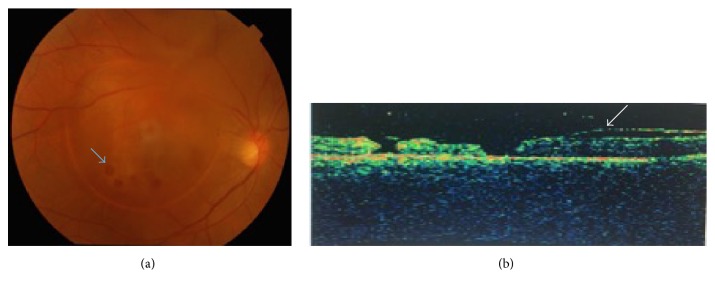
(a) Blue arrow indicates retinal hole. (b) White arrow indicates detached thickened ILM.

A 25-year-old male presented with sudden loss in vision of right eye (RE) following lifting heavy load. The presenting best corrected visual acuity (BCVA) in his RE was 20/200 and left eye (LE) was 20/20. Fundus evaluation of RE revealed well circumscribed dome shaped premacular haemorrhage about 3 disc diameters in size. Since the patient was very apprehensive and requested aggressive treatment, YAG laser hyaloidotomy/membranotomy was done with Zeiss Visuals® YAG III machine with a single shot of 8 mJ applied over inferonasal border of the subhyaloid haemorrhage. At 2-week follow-up, the premacular haemorrhage was noticeably absorbed but he developed 2 full thickness retinal holes near the inferotemporal border of premacular haemorrhage along with empty cavity as bubble over macula. At 4 weeks, the full thickness retinal holes increased to 5 in number (Figure 1(a)). Optical coherence tomography (OCT) showed 5 full thickness retinal holes with intact but thickened internal limiting membrane (ILM) detached in convex dome shaped configuration over macula (Figure 1(b)). Though there was significant resolution of haemorrhage, there was no improvement in his visual acuity. PPV with ILM peel with gas tamponade was done.

**Figure 2:**
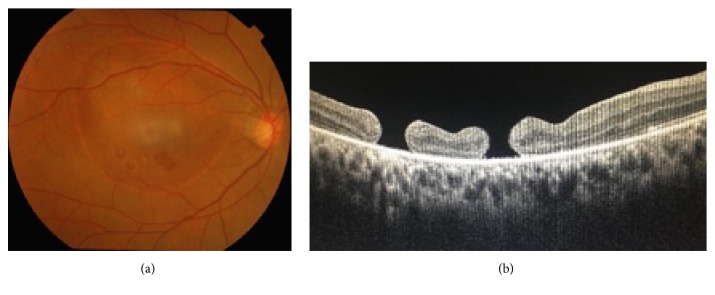


Intraoperatively, brilliant blue was used for staining, and the ILM was intact, but superficial sheen like dome shaped detachment suggestive of thickened ILM was present over the macular area. After PPV, though the fundus image shows persistence of empty bubble/cavity (Figure 2(a)), there was resolution of hyperreflective convex cavity in the post-PPV OCT (Figure 2(b)) with improvement in BCVA to 20/60. Six months after the onset, the BCVA, fundus examination results and OCT findings were stable.

## 3. Discussion

Valsalva retinopathy occurs in young healthy adults following a Valsalva maneuver. The prognosis for patients given a diagnosis of only Valsalva retinopathy is generally good. YAG hyaloidotomy is a relatively safe procedure with very good safety profile. It is noninvasive, inexpensive outpatient procedure and very effective treatment option for large subhyaloid haemorrhage in comparison to other treatment options like vitrectomy, pneumatic displacement. In the past, the application of Nd-YAG laser in the posterior segment was limited to releasing tractional vitreous bands and other pathologic vitreous attachments, especially in cases with tractional retinal detachment due to diabetic or sickle-cell retinopathy and other complicated retinal detachments. The high levels of energy (up to 1000 mJ) applied to treat these conditions could result in serious complications such as retinal and/or choroidal haemorrhage and retinal perforation. However no such events have been reported following YAG hyaloidotomy for subhyaloid haemorrhage, probably due to the fact that the energy used is very low and also the entrapped blood acts as a cushion protecting the underlying retina. To the best of our knowledge, the development of multiple retinal holes in the posterior pole following complete resolution of Valsalva retinopathy by YAG hyaloidotomy has not been previously reported in the literature. Kim et al. [1] reported a MH following vitrectomy for Valsalva retinopathy. Ulbig et al. [2] reported a MH identified after laser treatment. Xie et al. [3] reported lamellar MH associated with Valsalva retinopathy.

There are several possible mechanisms that could explain retinal hole formation following YAG hyaloidotomy.

First, the retinal holes could be due to the direct photo-disruptive effect of the YAG laser. The entrapped blood is believed to act as a cushion, dampening the disruptive impact of the ND: YAG laser burst. In small haemorrhages, the protective dampening effect may be insufficient.

Secondly, the thickened internal limiting membrane (ILM) observed after resolution of the premacular haemorrhage may have created a tangential traction on the retina.

Thirdly, the massive haemorrhage puts the ILM under high tension, which may make ILM hard to reattach to the retina as discussed by Zou et al. [4] and Meyer et al. [5]. The partial detached ILM on the surface of the retina may produce traction on retina.

In our case, the second and third hypothesis might explain the pathology of retinal holes since, during PPV, the ILM over previous subhyaloid/sub-ILM bleed was found to be partially detached. Moreover the visual acuity and retinal findings stabilized after PPV with ILM peel. After PPV, the fundus image shows persistence of empty bubble/cavity over the macula which might be due to pigmentary changes secondary to the entrapped sub-ILM bleed. Moreover, there is resolution of hyperreflective convex cavity in the post-PPV OCT. We need a long term follow-up to see if the cavity/bubble like appearance disappears in subsequent follow-up.

## 4. Conclusion

Multiple retinal holes secondary to Valsalva retinopathy have been rarely reported and their mechanism needs further understanding. This case illustrates the possibility of retinal hole formation for Valsalva retinopathy.

## References

[1] Kim K. Y. O., Yu S.-Y., Kim M., Kwak H. W. O. (2014). Macular hole formation after pars plana vitrectomy for the treatment of Valsalva retinopathy: a case report.

[2] Ulbig M. W., Mangouritsos G., Rothbacher H.-H., Hamilton A. M. P., McHugh J. D. (1998). Long-term results after drainage of premacular subhyaloid hemorrhage into the vitreous with a pulsed Nd:YAG laser.

[3] Xie Z.-G., Yu S.-Q., Chen X., Zhu J., Chen F. (2014). Macular hole secondary to Valsalva retinopathy after doing push-up exercise.

[4] Zou M., Gao S., Zhang J., Zhang M. (2013). Persistent unsealed internal limiting membrane after Nd:YAG laser treatment for valsalva retinopathy.

[5] Meyer C. H., Mennel S., Rodrigues E. B., Schmidt J. C. (2006). Persistent premacular cavity after membranotomy in valsalva retinopathy evident by optical coherence tomography.

